# Portfolio practices in the principal evaluation process: A qualitative case study

**DOI:** 10.1016/j.heliyon.2024.e39467

**Published:** 2024-10-18

**Authors:** Ahmed Mohammed Alkaabi, Asma Khaleel Abdallah

**Affiliations:** a Foundations of Education Department, United Arab Emirates University, Al-Ain, United Arab Emirates; b Educational Leadership Department, Sharjah Education Academy, Sharjah, United Arab Emirates

**Keywords:** Principal, Portfolio, Evaluation, School leaders, Supervisors

## Abstract

**Background:**

The portfolio is a vital component of the evaluation of principals and serves as a repository of evidence of achievements, skills, and capabilities. It facilitates the meticulous recording and documentation of experiences undertaken. Furthermore, the portfolio is instrumental in the refinement of conceptual ideas over time, while simultaneously offering a panoramic vista of a school's operational ethos and its alignment with professional benchmarks.

**Objective:**

This study delves into the practices surrounding the principal portfolio and the formative and summative evaluation of principals by their evaluating supervisors. It aims to uncover principals’ perceptions regarding the obligatory nature of portfolios and other evaluative methodologies throughout both assessments. In light of the pivotal role that portfolios occupy in the realm of professional development and evaluation for school principals, the Abu Dhabi Education Council has mandated the compilation of principal portfolios within public schools. This mandate requires a detailed accounting of principals' proficiencies, achievements, and developmental trajectories. Markedly, this exploration stands as one of a scant number of inquiries probing the application of portfolios by school principals, with a particular focus on the context of the United Arab Emirates. It is poised to enrich the discourse on the utility of portfolios as instrumental tools for fostering leadership acumen and evaluative competency within the spheres of educator and principal preparation over the preceding two decades. The critical examination of these practices is vital for a nuanced understanding of their operational dynamics, the efficacy and challenges of e-portfolios, the array of opportunities they unveil, the identities of the involved stakeholders, and their ultimate objectives.

**Methods:**

This study adopted a qualitative research approach to gather and analyze non-numerical data in the form of concepts, opinions, and experiences presented by principals and their supervisors within the Al-Ain School District (overseen by the Abu Dhabi Education Council)). The cohort of participants comprised nine individuals endowed with extensive portfolio experience, six principals and three cluster managers. All worked in public educational institutions and were meticulously selected based on predefined criteria. This study relied on several data collection methods to obtain participants’ perspectives on their experiences with portfolio practices, including face-to-face interviews and documents from their portfolios.

**Results:**

A thematic analysis of the participant data yielded five salient themes reflecting the experiences of the principals: (1) inconsistent expectations for portfolio evidence; (2) portfolios summoned in response to rising tensions at the summative evaluation; (3) portfolios promoting reflective practices in formative evaluation; (4) portfolios susceptible to manipulation or falsification; and (5) excessive time spent building and developing portfolios. In order to maximize the utility of portfolios, this result invites school principals to unleash the latent potential inherent in their actions by leveraging portfolios as mirrors for reflective leadership and ongoing self-assessment.

**Conclusions:**

This research delineates both practical and theoretical ramifications for the application of professional portfolios. It elucidates the nature of evidence principals ought to incorporate within their portfolios. Furthermore, it endeavors to better inform principals of the evidence collection process, including what artifacts and documentation to gather and how to do so.

## 1 Introduction

In the realm of educational leadership, the portfolio of a school principal serves as a pivotal mechanism for encapsulating their professional journey and efficacy. This portfolio, a curated collection of academic and professional documents, shines a light on a principal's competencies, educational background, qualifications, and experiences. Beyond showcasing technical skills and knowledge, this compilation reveals insights into the principal's character and dedication to educational excellence. While “portfolio” might traditionally evoke images of financial assets—ranging from stocks and real estate to bonds and mutual funds—the term, within an educational context, assumes a richer, more nuanced significance [1]. For school principals, their portfolio is not merely an inventory of achievements but a dynamic record of their growth and impact in the educational sphere. It encompasses comprehensive evidence of completed initiatives, feedback from stakeholders, progress in school management and leadership, strategies for professional development, and reflective insights into their performance and growth trajectory. Such portfolios are integral to the educational evaluation system and offer a holistic view of a principal's contributions and areas in need of enhancement. As such, they serve as a cornerstone for both accountability and continuous improvement in educational leadership [2].

The inception of portfolios in the field of education dates back to the late 1980s and early 1990s [3,4]. The portfolio tasks require students to curate collections that demonstrate their achievement of specific standards, progress, and accomplishments in one or more domains. Furthermore, professional portfolios, celebrated for their long-standing history among artists, writers, and architects [5], have found widespread application across pre-service and in-service teacher-education programs. These professional portfolios are pivotal for learning and professional development and represent structured compilations of evidence that highlight teachers’ optimal work and significant achievements across various contexts [[6], [7], [8], [9]].

There are four main types of portfolios that each have different purposes: a dossier portfolio, a training portfolio, a reflective portfolio, and a personal development portfolio. A dossier portfolio is a documented record that shows the achievements and work needed to enter a profession or program and gives a comprehensive overview of an individual's accomplishments. Next, a training portfolio, which is required during a learning program, shows the professional knowledge, skills, or competencies that an individual has gained. These features are often supported by reflective comments to explain the chosen evidence and usually follow a specific format to ensure the suitability of the evidence provided. Then, a reflective portfolio shows personal developments and achievements that aim for promotion and admission processes. Finally, a personal development portfolio, which is an evaluative and reflective record of professional growth over a long period, allows individuals to discuss and reflect on their activities and how they shaped their identity [10].

In 1999, the Interstate School Leaders Licensure Consortium delineated six core competencies vital for school leaders in the development and execution of the portfolio and other evaluation instruments. These competencies encapsulate the facilitation of a collective vision for learning, one that garners the endorsement of the school community and propels the achievement of all students. Furthermore, they highlight the imperative for fostering a school environment and an instructional program that are conducive to student learning and the professional development of staff. Additionally, the competencies include the obligation to ensure the effective management of the organization's operations and resources, thereby securing a safe, efficient, and effective learning milieu. School leaders are also charged with the duty of collaborating with families and community stakeholders, addressing the diverse needs of the community, and leveraging community resources in support of educational goals. Paramount among these competencies is the adherence to principles of integrity, fairness, and ethical conduct, alongside the capacity to comprehend, engage with, and exert influence over the broader political, social, economic, legal, and cultural landscapes to champion the welfare and success of all students [11].

The basic features of the standard portfolio began to be used by school leaders and administrators, which gave rise to the principal portfolio and became common practice in countries worldwide, including the United States. The principal portfolio is defined as a “collection of thoughtfully selected exhibits or artifacts and reflections of an individual's experiences and the ability to lead, and of the individual's progress toward and/or the attainment of established goals or criteria” [12]. The principal portfolio serves as an archival record of evidence and a tool that can be referred to and reflected upon to improve practice. Principal professional portfolios have sparked recognition as legitimate tools for professional development but have had mixed success [13]. This is because, among other reasons, the portfolio is a product of the individual learner. Consequently, over the last two decades, a growing number of mandates have targeted professional portfolio practices among affiliated universities or school districts in countries such as the United States and Australia [14]. Portfolios are often built gradually over time and can serve as a tool for planning and monitoring professional development by incorporating learning objectives, learning activities, and achievements, into the portfolio. A portfolio can be designed for the intended purpose of a specific school or university but may not necessarily align with other institutions [15].

Principals play a crucial role in driving improvement and growth in schools and remain the primary source of leadership influence. The principal portfolio process encourages reflection to help principals enhance their performance and leadership, which ultimately improves student achievement and teacher effectiveness. By modeling reflection and improved practice, principals can inspire teachers to also reflect on best practices with their students [14]. The evaluation of principals should accurately assess their competencies. Evaluation processes should be informed by best practices and research that contribute to successful schools. The development and implementation of an evaluation document should align with the expected proficiencies of principals [13]. Worldwide research on the efficacy of professional portfolio practices has also emerged, although largely in the area of pre-service portfolios [3,16]. Some existing research challenges the portfolio process and provides alternative perspectives, but few studies have investigated leadership portfolios worldwide [[17], [18], [19]], with none in the United Arab Emirates (UAE) [20].

Building upon this understanding, the present study focuses on the pivotal role of portfolios in the professional development and evaluation of individual school principals, specifically within the context of the Abu Dhabi Education Council (ADEC)'s mandate for public schools. ADEC's requirement for the development of principal portfolios underscores the significance of these documents as comprehensive collections that not only illuminate the skills and achievements of principals but also chart their progress throughout the year. This practice, established over nearly a decade, underscores the value of documenting accomplishments and developmental trajectories, a process deemed crucial for both the evaluation and professional advancement of school leaders. Given the scant research on leadership portfolios, especially within the UAE, this study seeks to fill a critical gap by exploring the implementation of these practices, the emergence of e-portfolios, and the various challenges and opportunities they present. Furthermore, it aims to shed light on the stakeholders involved in this process and delineate the overarching objectives of maintaining such portfolios. Through an in-depth examination of the perceptions and experiences of principals and their supervisors, this study endeavors to enhance our understanding of the efficacy of professional portfolios in fostering educational leadership excellence.

This study seeks to enhance our comprehension of how school principals in the United Arab Emirates perceive the use of portfolios throughout the academic year. The main overriding question of the study is: How do school principals view the utilization of e-portfolios as experienced and implemented throughout the academic school year in the United Arab Emirates? Several sub-questions were derived from this main question, including: What benefits and drawbacks do principals experience while using professional portfolios? What is the key role of portfolios in the summative evaluation process?

By exploring the processes, opportunities, and challenges of principal evaluation portfolios, alongside principals’ experiences in both formative and summative evaluations, this research aims to illuminate best practices for maximizing portfolio effectiveness, pinpoint areas for improvement to counteract potential drawbacks, and clarify their role in evaluation processes. Such exploration is crucial for educational leaders aiming to refine the methodology for principal evaluation. The ensuing discussion synthesizes pertinent literature on the demand for portfolios in the UAE, alongside the advantages and disadvantages associated with the evaluation process, thereby equipping educational leaders with insights necessary for enhancing the efficacy of educational leadership through informed portfolio evaluations.

## 2 Literature review

### 2.1 The need for portfolios in the UAE

Over the past two decades, the role of principals has evolved markedly, driven by policy and educational reforms aimed at boosting student achievement. This evolution is notably influenced by initiatives like the No Child Left Behind Act and Race to the Top [21]. The United Arab Emirates, particularly in the Emirate of Abu Dhabi, including regions like Al Gharbia and Al Ain City, has not been left behind in this shift. The Abu Dhabi Educational Council (ADEC), the region's educational regulatory authority, has been at the forefront of introducing, revising, and enforcing new professional standards for principals. ADEC has positioned these standards as fundamental to the principals' responsibilities, including leadership duties, beliefs, and attributes, as evidenced by research and practice.

Guided by Lynne Pierson's framework, principals were mandated to uphold certain standards pivotal to the advancement of their schools. These included articulating a coherent vision and mission, facilitating ongoing educator training for effective instruction, making decisions that prioritized organizational welfare, leading in professional development, and fostering robust relationships with community stakeholders to fulfill school objectives. Pierson further delineated strategies for the efficacious implementation of these standards by highlighting an evolving expectation for principals not merely to lead with instructional prowess but also to cultivate a school culture that propelled student success [22]. This shift in the expectations and roles of school leaders prompted leadership preparation programs to underscore the synergy between instructional leadership and school enhancement. In alignment with these changes, the ADEC offered a spectrum of training programs designed to endow principals with the requisite skills, knowledge, and values. Among these initiatives was the training for the development of high-caliber electronic portfolios (e-portfolios), which served as reflective of the professional standards principals demonstrated in their leadership and management roles over the academic cycle. These portfolios and e-portfolios emerged as critical facets of professional practice, instrumental in augmenting the leadership efficacy of school principals [23].

Building on this foundation, the integration of the principal's portfolio into their evaluation process signifies a strategic pivot towards more authentic and contemporary assessment methodologies, emphasizing the cultivation of 21st-century leadership skills. Such an approach is reinforced by a body of research that champions professional development, leadership enhancement, and self-assessment abilities. Furthermore, it highlights the importance of self-confidence, a readiness to embrace risks, active participation in professional dialogues, engagement with scholarly literature, and the setting of precise objectives that are in harmony with the overarching goals of both the school and district at large [13,18]. This nuanced evaluation strategy not only aligns with the transformative trajectory of educational leadership but also promises to enrich the practice by fostering a reflective and growth-oriented mindset among school principals.

### 2.2 Benefits and drawbacks of professional portfolios during the evaluation process

The literature is replete with studies that highlight the advantages of using professional portfolios. This prevalent method allows educators to reflect on their educational practices, which leads to increased self-awareness [24]. Furthermore, portfolios serve as invaluable tools during self-evaluation processes and in the establishment of objectives for practice modification [3,17]. The capability to retrospectively analyze one's past actions through various perspectives during reflection is crucial for leadership development. This process provides an opportunity to understand and interpret past behaviors deeply [25]. The significance of reflective learning was sharply articulated by Dewey, who noted, “We do not learn from experience. We learn from reflecting on experience” [26]. Additionally, effective leaders engaged in deep reflection to critically examine the dynamics of power and challenge underlying assumptions and practices [27].

Without regular reflection, the risk of poor decision-making increases. For instance, a school leader might misinterpret previous successes as indicators of infallibility and neglect alternative viewpoints or potential outcomes, which could lead to dire results [28]. This underscores the importance of pausing to reflect on significant events for realizing one's full leadership potential [29]. Reflection is vital for leadership enhancement, and it offers leaders unique insights that help in reinterpreting problems and analyze situations from various perspectives or gaining a better understanding of their leadership domain. It enables exploration, discovery, and the unravelling of connections between different life experiences.

However, achieving high levels of reflection independently is challenging for school leaders; they need to cultivate this skill. Gray and Bishop emphasized the necessity of both challenges and support to foster leadership growth [30]. Evidence from portfolios suggests that principals must be pushed beyond their comfort zones and learn how to learn [31]. Such learning experiences are augmented by continuous support from supervisors, who encourage reflective practices for leadership development [20,25]. Yet, educational experts warn that sustained leadership improvement is less about the portfolio itself and more about the ongoing development process and reflections on the included experiences [32].

Additionally, a principal portfolio can act as a genuine tool for documenting actions and providing evidence of the school's growth and achievements [33]. Portfolios are useful for both formative and summative evaluations of principals' professional growth. They record learning and experiences related to the principals' work, school action plans, new initiatives, curricula, data analyses, and student learning. In doing so, they capture efforts, progress, achievements, and future development plans. This provides a comprehensive view of the school's standing in relation to professional standards. Proponents of portfolios highlight its role in enabling a more authentic assessment of professional performance and fostering reflective inquiry, thereby contributing to continuous improvement [13].

During both formative and summative evaluations, the use of principal portfolios offers supervisors a detailed view into the leadership practices of principals. This allows them to verify the prioritization of student achievement and school success. The portfolio enriches the evaluation process with a base of knowledge about the school and fosters principals' ownership of their evaluations, which contrasts sharply with the traditional checklist approach [33]. It also encourages meaningful interactions between supervisors and principals, among principals themselves, and between principals and teachers. Research by Morris on electronic portfolios indicates that e-portfolios support feedback from various assessors and offer a comprehensive understanding of what is known, understood, and areas needing improvement [34].

While the benefits of portfolios are clear, their drawbacks cannot be overlooked. The creation of principal portfolios is notably time-intensive [35]. Many school-leaders report struggling to juggle their school duties with the demands of portfolio development. Principals often find themselves in a continuous cycle of self-reflection and growth, which requires significant time to manage. Moreover, the time evaluators and supervisors spend on reviewing these portfolios is substantial. Portfolios, although seen as a genuine form of assessment and a valuable learning tool, can sometimes become burdensome and lose their authenticity. The diversity and complexity of portfolios means they may not always truly reflect genuine development and run the risk of being more about presentation than substance [34]. There is a tendency for principals to selectively include achievements in their portfolios while omitting significant challenges, which leads to a portrayal that lacks authenticity and is overly flattering. This kind of portfolio can end up being superficial and highlights a tendency towards self-promotion and evasion of critical issues. Portfolios are also susceptible to manipulation, just like any other evaluation form, where the content can be curated to present a misleadingly favorable view [35].

The literature review suggests that the comprehensive nature of principal portfolios is a double-edged sword: while enabling principals to reflect deeply on their practices and demonstrate their leadership competencies in areas that might not be immediately apparent, the extensive documentation required can be overly time-consuming and detract from their core leadership responsibilities. Furthermore, the potential for misrepresentation and dishonesty within the portfolio underscores the challenges associated with ensuring authenticity and integrity in the evaluation process. This study delves into a decade of e-portfolio use as mandated by the ADEC and explores the experiences of individual principals and their cluster managers in the UAE. In this context, “cluster manager” refers to those responsible for overseeing and evaluating school principals. The term “cluster manager” is consistently used throughout this study to align with the context of the UAE educational system.

## 3 Methods

This study adopted a qualitative research methodology by gathering and analyzing non-numerical data to delve into concepts, opinions, and experiences [36]. The qualitative approach enabled the researchers to explore “how individuals interpret their experiences, how they construct their worlds, and the meanings they ascribe to their experiences” [37]. The research was structured around a case study design, which served as a methodological tool to gain a deeper understanding of specific individuals and to identify challenges by examining them in detail.

The case study method, with its distinct features and advantages, is particularly well-suited to this research due to its ability to investigate specific phenomena within their real-life contexts. By employing a diverse array of data sources, it facilitates a comprehensive examination that extends beyond the limitations of a singular perspective, which offers increased depth of understanding [38]. Moreover, as highlighted by Yin, a significant strength of the case study approach is its capacity for triangulating evidence through the incorporation of various data types—ranging from interviews and documents to artifacts and observations [39]. This methodological richness provides a more extensive evidence base than that typically available in conventional historical studies and allows for a nuanced exploration of complex issues.

The process of identifying a case and its units of analysis is fundamental to the case study method. Yin underscored the importance of clearly defining the case and the unit of analysis from the outset as crucial steps in formulating pertinent research questions [39]. In the context of this study, the schools within the Al-Ain School District, under the oversight of the Abu Dhabi Education Council, were selected as the cases, with a particular focus on school principals and their supervisors as the primary units of analysis. This choice underscores the intention to delve deeply into the experiences of principals in the creation and utilization of their portfolios during evaluation processes.

Employing the case study design enabled a detailed investigation into the principals' portfolio-related experiences, thereby enhancing the credibility and relevance of the research findings. This enhancement is achieved regardless of the sample size and is attributable to two key factors: the insights, relevance, and authenticity that emanate from the meticulously selected cases, and the application of rigorous analytical methods combined with the researcher's expertise. Through the careful delineation of the fundamental units of analysis and the strategic framing of research questions, the case study approach proves instrumental in unveiling the intricate dynamics at play in the principals' evaluation processes. All procedures involving human participants in this study were performed in strict compliance with the ethical standards of the Institutional Review Board (IRB) at the University of Georgia, under the ethics approval reference [IRB ID: STUDY00005027].

### 3.1 Participants

This study employed purposive sampling techniques to select participants and ensured a focus on individuals with relevant knowledge and experience, which is critical for the study's credibility [40]. This approach emphasized the selection of candidates based on their readiness, availability, and capability to share their experiences and perspectives both expressively and reflectively [41,42]. The researchers set specific selection criteria for two distinct groups: principals actively involved in ADEC's current evaluation processes, with a minimum of three years of evaluation experience, cluster managers with evaluative and supervisory duties, and a willingness to engage in reflective conversations about their practices. Cluster managers were chosen for their roles in supervising and evaluating principals within the same region. They were required to have at least three years of experience in these areas, and an openness to discuss their experiences.

The initial selection for the study included 15 candidates—10 principals and five cluster managers. However, after accounting for withdrawals and criteria-based exclusions, nine experienced participants remained: six principals and three cluster managers, all of whom were well-versed in portfolio development within public schools overseen by the Abu Dhabi Education Council (their demographics are detailed in Table 1). The recruitment process began with the acquisition of a list of principals from the Al-Ain school district, followed by email outreach to elucidate the study's goals and participation criteria. Those expressing interest and meeting the eligibility requirements were then furnished with detailed project information and an electronic consent form, which not only affirmed their informed and voluntary participation but also facilitated the logistical planning of interviews. These 2-h sessions with each of the nine participants, aimed at further understanding and identifying commonalities in data, were instrumental in achieving a comprehensive grasp of the topic through the process of data saturation. As a result, they enriched the study with valuable insights into the practices surrounding educational portfolio development.

**Table 1:** Demographic profile of research participants.

Participants^a^	Gender	Job Position	Years of Experience	School Level∗∗
Mousa	Male	Principal	20	Cycle I
Adam	Male	Principal	28	Cycle II
Saif	Male	Principal	16	Cycle III
Salwa	Female	Principal	20	Cycle I
Neeli	Female	Principal	25	Cycle II
Shaikah	Female	Principal	19	Cycle III
Julia	Female	Cluster Manager	4	Cycle I
Maya	Female	Cluster Manager	5	Cycle II
Benjamin	Male	Cluster Manager	7	Cycle III

a Pseudonyms were used instead of original names. ∗∗Cycle 1, Elementary; Cycle II, Middle; Cycle III, High School.

### 3.2 Data collection

This study utilized a variety of data collection methods to gather participants' insights on their experiences with portfolio practices, which emphasized face-to-face interviews and document analysis. The employment of multiple methods facilitated triangulation and ensured data saturation. Data collection occurred from March 2020 to February 2022, with delays due to the Covid-19 pandemic and the time required to secure approvals from authorities for interviews and document reviews. Interviews served as the primary avenue for understanding school principals' and cluster managers' engagement with portfolio practices [43]. These interviews were semi-structured and balanced flexibility with a focus on the central purpose of the research; every interview lasted approximately three hours. This format allowed conversations to progress naturally while keeping discussions aligned with the research objectives. The semi-structured nature also gave researchers the freedom to introduce new pertinent questions that enhanced or supplanted the original set as needed. This adaptability was critical for probing into particular facets of portfolio use more deeply. The interview protocol, specifically designed for this study's target groups, acted as a flexible guide and fostered a thorough exploration of each participant's narrative within their unique school context. At the beginning of each interview, researchers introduced themselves and clarified the interview's objectives. All sessions were electronically recorded using an IC recorder to ensure accuracy and reliability in transcription. Participants were encouraged to express themselves in the language they were most comfortable with (English or Arabic). This resulted in a more authentic representation of their viewpoints. To protect privacy, all identifying information was removed from the interview transcripts.

Document analysis was also integral to this case-study research and served both to corroborate findings from interviews and enrich the body of evidence. Documents are valuable in research as they remain unaffected by the researcher's presence or individuals' manipulative tendencies, which offer an unaltered view of the research setting [38]. With appropriate consent, various documents were collected, including emails, evaluation and supervision plans, and principals' portfolios. These documents were reviewed before and after interviews to deepen understanding of the portfolio process and inform the development of targeted interview questions. For example, an email directive from a cluster manager to a principal regarding portfolio preparation “only when necessary” prompted further investigation during interviews to unpack the implications of the term “necessary.”

### 3.3 Data analysis

The data analysis in this study was done carefully to extract useful insights from the combined dataset. Bryman and Burgess highlighted the vital role of data analysis in qualitative research and described it as a complex process that involved finding, defining, categorizing, theorizing, explaining, exploring, and mapping [44]. This thorough approach helps to unravel the intricate connections of phenomena that are part of human experiences. Data analysis was used as a central and comprehensive technique throughout the study, from the beginning to the final stages of writing the manuscript. This detailed examination of the data looked for repeated themes and patterns to help identify themes within the interview transcripts, which were then analyzed thematically to discover and report emerging patterns [45].

Thematic analysis is a methodological tool to organize qualitative data that allows researchers to divide information into emerging themes. These themes are helpful in answering the research questions posed by the study, thus fulfilling its underlying purpose [46]. Before starting the data analysis journey, the researchers familiarized themselves with the transcripts by reading them multiple times. This initial step was important for getting a deep understanding of the data available. They then coded the transcripts from each interview carefully and identified both similarities and differences in the experiences and stories shared. These individual codes were then systematically grouped according to their thematic relevance. To further confirm and support the findings, direct quotations from the principals and cluster managers were included as both verification tools and illustrative examples.

The study used triangulation and member-checking to improve its credibility. Triangulation enabled the examination of the data from different angles, looking for consistency across different data sources to affirm the study's credibility. The agreement between participants' interview responses and the collected documents––including professional development agendas, invitation forms, email correspondences, and portfolios––reinforced this validation process. Moreover, a member check was conducted to provide participants with the chance to review their interview transcripts. This step ensured that their perspectives and recounting of experiences were recorded accurately and faithfully.

## 4 Results

The thematic analysis technique and examination of emergent codes, derived from the combined data sources, yielded five predominant themes that addressed the research questions. These themes are summarized in the subsequent sections.

### 4.1 Inconsistent expectations for portfolio evidence

All participants recognized the professional portfolio as a significant component of the evaluation process. Principal Adam, for example, was aware of his duty to compile and organize data for his cluster manager's thorough examination of each evidence piece. Principals were tasked with creating a well-structured professional portfolio for evaluation by their assigned cluster manager, though such assessments were infrequently requested. Motivated by the opportunity to use their comprehensive portfolios for both evaluative purposes and to contend for local accolades like the Hamdan Bin Rashid Award for Distinguished Performance, the Khalifa Award, and other notable awards within the nation, some principals demonstrated exceptional enthusiasm.

An examination of the portfolios, along with their supporting documents and files, uncovered an extensive collection, often exceeding hundreds of pages. The inclusion of items such as school improvement plans, teacher evaluations, professional development forms, student data and work samples, survey responses, and staff meeting agendas showcased the principals’ adherence to the standards of effective leadership. Nonetheless, several principals encountered difficulties in assembling “compelling evidence.” Shaikah pointed out that these challenges arose from the inconsistent and varied criteria that cluster managers applied in determining what constituted acceptable and reliable evidence. Such disparities in expectations among cluster managers could potentially undermine the integrity of the principal evaluation process. She elaborated on the following points:

Every cluster manager has [a] different understanding of the meaning of acceptable evidence. Two years ago, I remember one of the pieces of evidence I presented was rejected by my former cluster manager when it was accepted last year by this current cluster manager … Besides, we were given a very basic orientation on documenting, but with no adequate details to guide us in amassing valid evidence.

Principal Shaikah also faced challenges due to collaborating with a new cluster manager biennially, each harboring their own interpretation of what constituted acceptable evidence. She described this experience as “confusing and upsetting.” Similarly, Principal Mousa expressed difficulties in compiling evidence for her portfolio, detailing her struggles with the process:

Cluster managers have to come to terms with finding a unified definition of compelling evidence backed up with clear examples … to provide follow-ups with their principals to ensure they [were] on the right track of compiling their portfolio evidence. Otherwise, this practice is meaningless … you know! Everyone works [in a] different direction.

Principal Neeli voiced concerns over the challenges principals encountered when cluster managers persisted in demanding evidence without establishing specific criteria. The inconsistency in defining valid evidence among cluster managers led to confusion and disarray during the summative assessments. This lack of a uniform definition for valid evidence complicated the evidence collection process for principals’ portfolios due to ambiguous evaluation standards.

All participants in cluster management agreed that the evaluation criteria could have multiple meanings. For example, cluster manager Maya said, “it's not a given,” meaning that a principal might satisfy her success criteria but not those of another cluster manager using the same framework. Cluster manager Benjamin agreed, pointing out the flexible nature of the criteria, which could differ a lot among cluster managers. Despite these difficulties, cluster manager Maya highlighted the importance of having meetings and workshops to agree on the definitions and expectations of evaluation standards. However, clarity required more than just agreement; it also needed clear expression and detailed examples covering all aspects of the evaluation criteria. From these observations, the researchers concluded the vital role of professional educational workshops in improving the knowledge and comprehension of both principals and cluster managers. Such workshops are useful in creating professional portfolios and explaining the kinds of evidence that are suitable for evaluation purposes.

### 4.2 Portfolios summoned in response to rising tensions at the summative evaluation

Nearly all principals and cluster managers acknowledged a shift in their electronic portfolio (e-portfolio) practices. When interviewed, principal Neeli revealed that although she initially found the portfolio to be beneficial, its significance diminished in the later stages of the evaluation process. She highlighted the importance of portfolios for documenting professional learning and development, as they offered a comprehensive overview of skill progression. However, she also noted a lack of interest from cluster managers in reviewing the entire portfolio, which led her to feel demotivated about creating detailed portfolios that were seldom examined in depth. Moreover, due to the portfolios' extensive nature, they were primarily utilized as evidence only in instances of disagreement.

Principal Salwa shared several emails from her cluster managers over three consecutive years concerning the organization of the final evaluation visit, with advice to “make the portfolio ready, just in case we need to look at it.” This suggested that the portfolio was considered useful primarily in cases of dispute. Similarly, principal Saif received an email from his cluster manager stating, “Do not worry about portfolio check scrutiny … I will ask if necessary, if we have a debate.” This illustrates the portfolio's role in resolving disagreements during the summative evaluation process. This sentiment was consistent across interviews, with nearly all participants concurring that the portfolio served as conclusive evidence in situations where there was a discrepancy between the principal's self-evaluation and the cluster managers' assessment. Cluster manager Benjamin also shared this view as he affirmed in the following quotation:

We would go and sit down with the principal, and we would take a look at it [the initial evaluation]. If there was any difference between what they said and what I believed, we talked about it and examined the evidence. It would be great if we came to an agreement. If we did not agree, then we rechecked the evidence. And if I still did not agree, they might accept that … but if they did not accept that, then it was fine. We looked through your evidence as well. We discussed it together.

The portfolio was regarded as a crucial tool for resolving tensions arising from discrepancies between the principal's self-evaluation and the cluster manager's initial assessment. Cluster manager Benjamin stated, “I would request evidence from the portfolio in such cases, even if I was unsure whether the principal had it.” This indicates that cluster managers had adequate time to assess each principal along the developmental continuum and offered mentoring and guidance to help them meet expectations, particularly when they were closely monitored throughout the year. Reflecting on cluster managers not necessarily checking the portfolio page by page, cluster manager Julia stated, “Cluster managers are more into examining data in [an] in-depth manner through weekly visits … So, this makes it more feasible and convenient in making [an] evaluation of the principal's evaluation.” This highlights that the involvement of cluster managers and administrative teams in the formative evaluation process reduces the reliance on e-portfolios in practice. All cluster managers acknowledged that they only asked for a portfolio when tensions arose, as their weekly visits were deemed sufficient for assessing the school's function and the principals' work. Despite the less frequent review of e-portfolio content, some school principals favored including them in the evaluation process.

### 4.3 Portfolios promoting reflective practices in the formative evaluation

Interviews with principals and cluster managers underscored the utility of portfolios when crafted authentically. Specifically, a portfolio should encapsulate deliberate actions and solutions aimed at enhancing the school's direction, with an emphasis not solely on student development but also on the growth of teachers and staff. Principal Mousa emphasized that portfolios ought to accurately represent the school's ethos and the educational programs it hosts. Constructing portfolios in such a manner has the potential to broaden the application and relevance of electronic portfolios, both now and in the future. Reflecting on her eight-year journey with e-portfolios, principal Shaikah shared her insights and experiences:

As a principal who uses a portfolio as a means for recording and documenting my actions and reflecting upon various experiences … I find this kind of process helpful in constructing a framework for self-assessment and continual reflections. In doing so, I could look back on what I would do differently, what worked, and what didn’t! I might even archive these things over into the next year so if something did not work, I know it is there!

The portfolio's usefulness in keeping the focus on school priorities was clearly shown by the accounts from principals and cluster managers. Principal Adam said the portfolio was “a mirror that helped him examine the situations encountered and decisions made and then reflect it back to him to generate a better decision.” He also emphasized the importance of the reflective inquiry process, which helped him in “replaying the situations over and over” and “reflecting on them” carefully to improve future actions. A common theme among principals in terms of reflective practices within the portfolio was the idea of doing a retrospective analysis to find better solutions for past problems. This process allowed them to reevaluate their “actions to see if there [was] a better, more effective way of handling situations” involving stakeholders such as ADEC officials, teachers, students, and parents. However, principal Neeli noted an important condition: “Reflection should be a habitual practice that is ongoing … and with the assistance of colleagues [administrative teams and teachers] and cluster managers if needed, not an episodic thing.”

In another area of reflection, most participants supported ‘reflection-in-action,’ where principals examined scenarios inwardly and created possible solutions. As they reviewed artifacts showing specific situations, they took part in a reflective process to come to understand what they knew, what happened, why it happened, how it could have been handled differently, and what lessons could be learned. This involved checking their beliefs, assumptions, goals, attitudes, and competencies to develop new understandings and insights. Aside from personal reflection and self-evaluation, the portfolio also encouraged discussions with colleagues, especially when a major decision needed collective knowledge and attention. Principal Saif shared his thoughts on such experiences:

Many brilliant ideas and decisions come from the teachers at my own school. Without a question, it is important to communicate with one another, share ideas, and build capacity. Principals, regardless of their network and counterpart principals to consult with, have a staff who are talented and want to be heard and engaged in many ways in the school development … And the portfolio is an opportunity, you know … I always seek consultation from vice principals, head departments, teachers … Sometimes it goes down to asking students! By the way, the composition of [the] portfolio is not [the] solitary job of the principals, but it is collective work for [the] collective improvement of our school.

Principals have underscored the significance of school administrators and teachers collaboratively creating portfolios to showcase the school's learning and growth, supported by tangible evidence. Principal Saif noted these portfolios could illustrate the school's and its community's overall advancement. Similarly, principal Salwa stressed the importance of involving additional staff members in creating these portfolios. She argued that confining this experience solely to principals could compromise their roles and advocated instead for a collective effort to amass a comprehensive and excellent set of portfolio materials. This approach, she suggested, would more accurately reflect the school's reality and emphasize its continuous improvement.

Conversely, cluster managers hold varying opinions on the effectiveness of principals' portfolios. Some observe that they do not always capture the full scope of a principal's capabilities. For instance, Julia observed that some principals treated the creation of their electronic e-portfolios as mere obligations, while others recognized its value as a reflective tool and leveraged it to enhance their schools' growth. Similarly, Benjamin shared an instance of a principal who reached out for a meeting to review the school's progress over the first and second years, which underscored the varied approaches to portfolio development and use.

I was satisfied when I found an email from my principal in my inbox. She was reaching out for a quick meeting to double-check some information and to see for ourselves if there had been real progress. I made my way to her office the very next day. There, she took me through the portfolio, with a keen eye on the analysis and feedback shared from the first term to the second. It opened up a rich conversation and gave her the space to think over the methods that brought about this change. More importantly, it sparked thoughts on what she would keep the same or tweak as we headed into the third term.

Maya emphasized that the portfolio could serve as a valuable resource not only for facilitating communication between principals and their cluster managers but also among members of the school leadership team. It offered a platform for them to engage in introspection regarding their practices, conduct self-assessments of their actions, and implement the necessary enhancements. However, Maya shared insights from her experience indicating that this ideal utilization was not universally adopted. She pointed out that, while some principals and their teams proactively engaged in reflective practices to acquire a comprehensive understanding of their school's position with sincere interest and even solicited visits to augment these reflective efforts, there existed a contrasting group. This latter group, regrettably, undertook the creation of portfolios in a perfunctory manner, driven solely by the obligation of compliance. In this case, they failed to recognize and leverage the portfolio's full potential for fostering reflection and facilitating improvement.

### 4.4 Portfolios susceptible to manipulation or falsification

The participants acknowledged that the portfolio was made with misconduct, which made it invalid and unrepresentative of the schools—it was more like a fantasy. Principal Mousa exposed the current wrongdoing and said that the portfolio should not be just a document with embellishments and inventiveness but must state the school's progress accurately and include the issues and disputes that need to be resolved. He stated that most school principals made portfolios that had excessive documentation and lacked pertinent information on overall growth and development. This was unfair to the practice of portfolios, he claimed. Likewise, principal Adam shared his experience:

If your fake portfolio is showing the evaluator a massive improvement of your school, and you can’t get your head [around] it because it was never actually there, how will you feel? Pretty deprived, I would think! Cluster managers are not fooled by any of this because they [are] weekly on the school grounds and know the sort of improvements made and what is going on! But I heard this malpractice going [on] among school principals!

Principal Mousa emphasized the utility of portfolios when constructed authentically and accurately. He asserted that they should encompass not only student development but also the professional growth of teachers and personnel, thus mirroring the school's essence and the educational endeavors within. On a similar note, principal Neeli illustrated the potential for last-minute alterations in portfolios by showcasing sections on parent communication that had been modified. She acknowledged the efficacy of regular oversight by ADEC and cluster managers in mitigating such discrepancies. Cluster manager Maya critiqued portfolios for their lack of genuine content and suggested they often contained filler material that was irrelevant to the school's actual operations, a sentiment echoed by Benjamin who noted inconsistencies between portfolios and observed practices. Seeking to solve this issue, principal Julia aimed at a thorough understanding of school dynamics as a proactive strategy to aligning documentation with reality. Benjamin and Maya both advocated for direct comparisons and on-site evaluations as methods to ensure portfolio accuracy, highlighting the need for portfolios to reflect genuine school performance and practices.

Cluster manager Benjamin argued against the sole reliance on portfolios for authenticity when she suggested that without correlating documentation to actual school practices, the portfolios' integrity could be compromised, as evidenced by disparities he noted between documented achievements and classroom realities. In contrast, Maya utilized specific techniques, such as scrutinizing discrepancies between reported grades and classroom observations, to ensure alignment between portfolio documentation and the actual school environment. Her approach, which was underscored by frequent school visits and meticulous supervision, exemplified a method to counteract the superficiality of portfolios. This strategy not only highlighted the importance of direct oversight but also reaffirmed the critical role of cluster managers in discerning the true dynamics of school operations beyond mere documentation. Through these narratives, the discussions around portfolios revealed a spectrum of challenges and strategies while underscoring the complex interplay between documentation, oversight, and the authentic representation of school practices.

### 4.5 Excessive time spent building and developing portfolios

One challenge that principals and cluster managers faced with their portfolio experiences was how much time they took. One participant called the portfolios “prisons of time,” and a few others said something similar. They needed a lot of time to put together, as well as careful and deliberate practice, to achieve quality assurance; otherwise, they would look like just work stuffed into folders. Principal Saif shared his story of creating his portfolio:

It is too much time. It requires hard work and is very time-consuming, and frankly, there is no time for it in the daily race. We [principals] barely hold our breath and work on school routines and other tasks. But it is mandatory. We must do it anyway!

The portfolio was also a concern for some participants because of the time it demanded. For some, the portfolio was burdensome and added to their already heavy workload. To make the portfolio more beneficial and less time-consuming, principal Mousa said that he increased collaboration among administrative teams and different departments that worked together to compile their portfolios and avoid leaving any principal alone in the process. Principal Shaikah explained how teamwork could make the portfolio creation faster, especially when it involved administrators and teachers who were really interested and eager to do it. Principal Mousa described his experience with portfolios as he delegated tasks to department heads and experienced teachers:

There is no doubt [the] portfolio takes much time, but I am looking at the bright side and for the school’s benefit. I divide the work across departments and teams, and according to their experience. I have a team analyzing exam data … a team to monitor progress and update … a team checking initiatives … and there is always [a] certain date to visit the portfolio to ensure all things are in place, so we are on track and not lagging!

Some principals also said they kept their portfolios updated regularly in case their cluster manager asked to see them during their routine visits. Cluster manager Benjamin shared that when he began his supervisory role, he constantly checked the principals' portfolios during school visits. But he soon stopped doing that after finding out that the time for supervisory visits was precious and should not be wasted on trivial tasks like this. “That is what you do outside of school, and I can electronically verify it [the portfolio], if necessary,” he stated. Moreover, the shift from a conventional portfolio to an electronic portfolio can not only alter how principals and cluster managers create, update, access, link, and process performance information but also save a lot of time.

## 5 Discussion

The displeasure among principals concerning the ambiguity of rubrics and the conflicting interpretations by cluster managers notably complicated the portfolio collection process. Principals reported varied interpretations and guidance for identical criteria by different cluster managers, adversely impacting both evidence gathering and their performance evaluations. Despite the critical need, research focusing on the divergence in supervisors' interpretations of evaluation criteria remained scarce. However, some studies underscored the importance of achieving a consensus and harmony between supervisors and principals on portfolios' overarching goals, methods, and objectives to mitigate unintended detrimental impacts on the review process [47,48]. This approach could simplify and elucidate the evidence collection phase.

A noticeable shift in portfolio practices among principals and cluster managers was observed; whereas portfolios once underwent comprehensive reviews, they were now referenced primarily in disputes over initial evaluations. Although specific research on this shift was limited, existing studies suggested a pivot towards using portfolios as evaluative tools through shared artifacts and reflections [33], enhancing communication, and providing clearer insights into principals' performances during evaluations [13]. Additionally, a strong connection between portfolios and reflective practice in formative evaluation was identified, which advocated for portfolios to foster continuous reflection rather than serve solely for summative purposes. Such reflection allowed for a comprehensive review of principals' beliefs, values, and practices, crucial for future planning and improvement. Without ongoing reflection, portfolio creation was rendered ineffective, yet it was instrumental in encouraging principals to reflect on and enhance their leadership skills [14,29,31].

Manipulations within portfolios that potentially undermined evaluation integrity were also highlighted, with corroborating evidence from other research [34,35]. The reliability of stored evidence, and its validity as supportive documentation, was questioned [28]. To counter misuse, portfolios were suggested to function as a means for cross-referencing and affirming principals' performance, rather than as revered collections without critical scrutiny [14,38,49,50]. Discussions with study participants revealed the significant effort and time investment portfolios demanded and emphasized the need for meticulous compilation by school administrators. Continuous supervision by cluster managers throughout the year effectively guided and developed principals to meet expected standards, as supported by findings that portfolio compilation was an extensive, labor-intensive process [14,34,35]. To facilitate this, administrators often leveraged their teams for assistance in developing portfolios, from fact-finding to presenting strategic initiatives.

## 6 Conclusion

This qualitative study suggested implications for practice and future research. The most dominant practice voiced by participants regarding the use of portfolios was the reflective practice associated with it. According to the principals, this reflection encouraged them to constantly assess the effectiveness and impact of their leadership practices and beliefs. They became much better able to see connections between past and future actions and therefore made more calculated and thoughtful decisions about future actions. Furthermore, the portfolio process involves continuous assessment, including both formative and summative evaluations. However, to facilitate this process, continuous training provided by cluster managers for school principals on reflective practices is crucial for developing leadership skills and assisting principals in staying on track. Cluster managers are required to regularly remind principals of the reflective aspects and provide clear examples to make a profound difference in their use of portfolios. The buy-in for using portfolios as learning tools, where principals monitor their progress, assess their initiatives, school term reports, and performance regularly throughout the academic year, is more crucial than compiling all documents at the end of the summative evaluation.

Principals have voiced concerns regarding the acceptability of evidence to collect, as each cluster manager may have a different perspective on what constitutes acceptable evidence for inclusion in the portfolio. Thus, there is a need for clear articulation and ample examples to cover every aspect of the evaluation criteria. Organizing professional workshops can contribute to raising awareness and understanding among principals and cluster managers, which in turn would help them as they create professional portfolios and clarify the types of acceptable evidence relevant to the evaluation criteria. Professional development conducted by cluster managers for principals can also help clarify and unify practices by ensuring any confusion regarding unspoken aspects of the portfolio during formative and summative evaluations is addressed. Likewise, it is highly recommended that cluster managers conduct ongoing weekly visits and observations to school principals to provide a more accurate assessment of performance and to prevent portfolio manipulation or falsification that could misrepresent the school's true performance. Regular visits can foster a culture of transparency and collaboration between principals and cluster managers, encouraging open communication and mutual understanding, with no significant surprises at the summative evaluation. Moreover, these regular visits can create new learning opportunities for principals to consult with cluster managers about any struggles and issues they might face.

Finally, the principals engaged key figures in a joint collaborative effort to create the portfolio. This is because creating a portfolio was time-consuming and necessitates dedication from the principal, as the principal's work reflects the efforts of the entire school. Participants in the study highlighted the importance of involving experts within the school in compiling various documents for the portfolio, such as school data, initiatives, committees, and professional development activities. Furthermore, the use of electronic portfolios facilitated document retrieval and allowed for swift modifications, in contrast to the traditional use of portfolios, where reviewing pages and examining specific documents from the portfolio binder might be time-consuming.

This qualitative study has certain limitations. One significant limitation is the small number of participants, which may affect the generalizability of the findings and may not fully capture the diversity of perspectives within the broader population. However, despite this limitation, the participants provided rich, detailed, and valuable insights into the practices surrounding the principal portfolio. Additionally, this research required extensive time for data collection, not only due to the COVID-19 pandemic but also because of the time needed to obtain approval from authorities to access and review documents related to the portfolio.

Future research may broaden this study to encompass a larger scale and employ a quantitative approach to investigate a wider sample across all seven emirates of the United Arab Emirates. In addition, a mixed-methods study could compare novice and veteran principals' experiences with portfolio use. Exploring how portfolios enhance leadership skills among school principals could be the focus of a qualitative study. This multi-faceted research approach would offer comprehensive insights into the effectiveness of portfolios. Ultimately, these studies could contribute significantly to understanding and improving portfolio use in educational leadership.

Portfolios play a pivotal role in the life of a school principal and serve as a dynamic tool for reflection, self-assessment, and professional development. They not only showcase the achievements and progress of principals but also provide a platform for ongoing improvement and growth. The insights gained from this study underscore the significance of authentic and reflective practices in portfolio creation. They further highlight its value in enhancing leadership skills and promoting school improvement. As principals continue to utilize portfolios as a means of documenting their journey and impact, they are better positioned to lead their schools effectively and contribute significantly to the educational landscape.

## CRediT authorship contribution statement

**Ahmed Mohammed Alkaabi:** Writing – review & editing, Writing – original draft, Supervision, Methodology, Investigation, Data curation. **Asma Khaleel Abdallah:** Writing – review & editing, Writing – original draft, Supervision, Methodology, Investigation, Data curation.

## Data availability statement

The data related to this study is not in a public repository because it contains sensitive information.

## Declaration of competing interest

The authors declare that they have no known competing financial interests or personal relationships that could have appeared to influence the work reported in this paper.

## References

[1] Driessen E., Jan Van T., Cees Van Der V., Val W. (2007). Portfolios in medical education: why do they meet with mixed success?. A systematic review: Med. Educ..

[2] Driessen E., Jan van T. (2013).

[3] Berrill B. (2002). Professionally Speaking: the Magazine of the Ontario College of Teachers.

[4] Rolheiser C., Schwartz S. (2001). Pre-service portfolios: a base for professional growth. Can. J. Educ..

[5] Glatthom A.A. (1996).

[6] Oner D., Adadan E. (2016). Are integrated portfolio systems the answer? An evaluation of a web-based portfolio system to improve preservice teachers' reflective thinking skills. J. Comput. High Educ..

[7] Rogers C., Scales R.Q. (2013). Preservice teachers' perceptions of teacher leadership: is it about compliance or understanding?. Issues Teach. Educ..

[8] Pereira Í.S.P., Parente M.C.C., da Silva C.V. (2016). Guided portfolio writing as a scaffold for reflective learning in in-service contexts: a case study. Teach. Dev..

[9] Kloser M., Borko H., Wilsey M., Rafanelli S. (2022). Leveraging portfolios in professional development for middle school science teachers' assessment and data use practice. Sci. Educ..

[10] Smith K., Harm T. (2003). Clarifying different types of portfolio use. Assess Eval. High Educ..

[11] Murphy J., Shipman N. (1999). The interstate school leaders licensure consortium: a standards-based approach to strengthening educational leadership. J. Person. Eval. Educ..

[12] Brown G., Irby B.J. (2001).

[13] Marcoux J., Brown G., Irby B.J., Lara-Alecio R. (2003). Paper Presented at the Annual Meeting of the.

[14] Mestry R., Schmidt M. (2010). Portfolio assessment as a tool for promoting professional development of school principals: a South African perspective. Educ. Urban Soc..

[15] Driessen E. (2008).

[16] Riggs I.M., Sandlin R.A. (2000). Teaching portfolios for support of teachers' professional growth. National Association of Secondary School Principals Bulletin.

[17] Dietz M.E. (2001). Designing the school leader's portfolio. Arlington Heights, IL: Skylight.

[18] Dixon K. (2002). http://www.aare.edu.au/02pap/dix02119.htm.

[19] Schwartz S. (2003). Professional portfolios: a tool for reflective practice. OPC Regist.: The Magazine for Principals and Vice-Principals Across Ontario.

[20] Alkaabi A.M. (2021). A qualitative multi-case study of supervision in the principal evaluation process in the United Arab Emirates. Int. J. Leader. Educ..

[21] Reid D.B. (2019). Shared leadership: a comparative case study of two first year US principals' socialization around teacher evaluation policy. Educ. Manag. Adm. Leader.

[22] Pierson L. (2011). Education in the UAE: Current Status and Future Developments, Abu Dhabi, Emirates: Emirates Center for Strategic Studies and Research.

[23] Montecinos C., Flessa J., Valenzuela J.P. (2022). Pathways to the school principalship: an international scoping review. Educ. Manag. Adm. Leader.

[24] M Alkaabi A., Almaamari S.A. (2020). Supervisory feedback in the principal evaluation process. Int. J. Eval. Res. Educ..

[25] Samaa G., Khadeegha A. (2019). Exploring teacher perceptions of using e-portfolios in public schools in the United Arab Emirates. Int. J. Educ. Literacy Stud..

[26] Dewey J. (2019).

[27] Densten I.L., Gray J.H. (2002). Leadership development and reflection: what is the connection?. Int. J. Educ. Manag..

[28] Avolio B.J. (2008).

[29] Gray C., Bishop Q. (2009). Leadership development: schools and districts seeking high performance need strong leaders. J. Staff Dev..

[30] Chikoko V., Naicker I., Mthiyane S.E. (2011). Leadership development: principals' portfolio as an instrument for change. Educ. Change.

[31] Russo A. (2004). Evaluating administrators with portfolios. Sch. Adm..

[32] Du Plessis L.A., Koen I. (2005). Portfolio assessment of information technology students at the University of Technology: a case study. Educ. Change.

[33] Hargreaves A., Earl L., Schmidt M. (2002). Perspectives on alternative assessment reform. Am. Educ. Res. J..

[34] Morris J., Crawford C., Davis N., Price J., Weber R., Willis D. (2003). Proceedings of SITE 2003--Society for Information Technology & Teacher Education International Conference.

[35] Ontario Principals’ Council (2002).

[36] Coe R., Waring M., Hedges L.V., Ashley L.D. (2021).

[37] Merriam S.B., Tisdell E.J. (2016).

[38] Patton M.Q. (2015).

[39] Yin R.K. (2014).

[40] Creswell J., Plano-Clark V. (2011).

[41] Bernard H.R. (2002).

[42] Spradley J.P. (1979).

[43] Seidman I. (2012).

[44] Bryman A., Burgess R.G. (1994).

[45] Maguire M., Delahunt B. (2017). Doing a thematic analysis: a practical, step-by-step guide for learning and teaching scholars. All Ireland Journal of Teaching and Learning in Higher Education.

[46] Braun V., Clarke V. (2008). Using thematic analysis in psychology. Qual. Res. Psychol..

[47] Oudkerk P., Andrea A., Debbie C., Jaarsma E., Driessen E., Marjan J.B. (2020). Student perspectives on competency-based portfolios: does a portfolio reflect their competence development. Perspectives on medical education.

[48] Davis S., Kearney K., Sanders N., Thomas C., Leon R. (2011). https://www.wested.org/online_pubs/resource1104.pdf.

[49] Goldring E., Cravens X., Murphy J., Porter A., Elliott S., Carson B. (2009). The evaluation of principals: what and how do states and urban districts assess leadership?. Elem. Sch. J..

[50] Al Maktoum S.B., Al Kaabi A.M. (2024). Exploring teachers' experiences within the teacher evaluation process: a qualitative multi-case study. Cogent Education.

